# The walnut *JrVHAG1* gene is involved in cadmium stress response through ABA-signal pathway and MYB transcription regulation

**DOI:** 10.1186/s12870-018-1231-7

**Published:** 2018-01-22

**Authors:** Zhenggang Xu, Yu Ge, Wan Zhang, Yunlin Zhao, Guiyan Yang

**Affiliations:** 1 Hunan Research Center of Engineering Technology for Utilization of Environmental and Resources Plant, Central South University of Forestry and Technology, 498 Shaoshan South Road, Changsha, Hunan Province 410004 China; 2 School of Material and Chemical Engineering, Hunan City University, 518 Yingbin Road, Yiyang, Hunan Province 413000 China; 3 College of Forestry, Hubei University for Nationalities, 39 Xueyuan Road, Enshi, Hubei 445000 China; 4 Laboratory of Walnut Research Center, College of Forestry, Northwest A & F University, Yangling, Shaanxi 712100 China

**Keywords:** Heavy metal stress, *JrVHAG1* gene, ABA-signal pathway, Promoter, MYB transcription factor

## Abstract

**Background:**

Vacuolar H^+^-ATPase (V-ATPase) is a vital protein complex involved in abiotic stress response in plants. The G subunit of *Juglans regia* (*JrVHAG1*) was previously identified as a drought tolerance-related gene involved in the ABA (abscisic acid)-signal pathway. Heavy metal stress is becoming a major detriment for plant growth, development, and production. In order to understand the role of *JrVHAG1*, the potential function mechanism of *JrVHAG1* exposed to CdCl_2_ stress was confirmed in this study.

**Results:**

Transcription of *JrVHAG1* was induced by ABA and increased to 58.89-fold (roots) and 7.38-fold (leaves) and by CdCl_2_ to 2.65- (roots) and 11.42-fold (leaves) relative to control, respectively. Moreover, when treated simultaneously with ABA and CdCl_2_ (ABA+CdCl_2_), *JrVHAG1* was up-regulated to 110.13- as well as 165.42-fold relative to control in the roots and leaves, accordingly. Compared to the wild type (WT) Arabidopsis plants, the transgenic plants with overexpression of *JrVHAG1* (G2, G6, and G9) exhibited increased seed germination rate, biomass accumulation, proline content, and activities of superoxide dismutase (SOD) and peroxidase (POD) under ABA, CdCl_2_, and ABA+CdCl_2_ treatments. In contrast, the reactive oxygen species (ROS) staining, malondialdehyde (MDA) content, hydrogen dioxide (H_2_O_2_) content, as well as electrolyte leakage (EL) rates of transgenic seedlings were all lower than those of WT exposed to ABA, CdCl_2_ and ABA+CdCl_2_ stresses. Furthermore, a 1200 bp promoter fragment of *JrVHAG1* was isolated by analyzing the genome of *J. regia*, in which the *cis*-elements were identified. This *JrVHAG1* promoter fragment showed expression activity that was enhanced significantly when subjected to the above treatments. Yeast one-hybrid assay and transient expression analysis demonstrated that *JrMYB2* specifically bound to the MYBCORE motif and shared similar expression patterns with *JrVHAG1* under ABA, CdCl_2_ and ABA+CdCl_2_ stress conditions.

**Conclusions:**

Our results suggested that the *JrVHAG1* gene functions as a CdCl_2_ stress response regulator by participating in ABA-signal pathway and MYB transcription regulation network. *JrVHAG1* gene is a useful candidate gene for heavy metal stress tolerance in plant molecular breeding.

**Electronic supplementary material:**

The online version of this article (10.1186/s12870-018-1231-7) contains supplementary material, which is available to authorized users.

## Background

Heavy metals include micronutrients (such as Fe, Mn, and Mo), trace elements (such as Cu, Zn, Ni, and W), and stress factors (such as Cd, Pb, Hg, Ag, and U) that are toxic to plants [1]. Heavy metal stress not only leads to decrease in plant seed germination, plant growth, or increase of reactive oxygen species (ROS) accumulation and cell death but also induces chlorosis, necrosis, and turgor loss, even modifies the protein profile [2, 3]. More importantly, enrichment of heavy metals in plants and animals causes serious harm to the food chain and human health. Therefore, adequate understanding of the regulatory mechanism and screening the vital functions of genes from corresponding plants are vital to understand plant heavy metal stress responses.

Among the stress related heavy metals, Cu, Zn, Pb, Ni, Se, Cr, and Co were moderately enriched, whereas Sb and cadmium (Cd) were extremely highly enriched in the majority of soil samples, therefore, Cd is regarded as one of the most phytotoxic heavy metals [4, 5]. In soil, Cd contamination could severely affect the performance of agricultural fields [6]. Cd toxicity in agricultural soil has received significant attention because of its high penetration in the food chain and its toxicity to humans [7]. Thus, characterization the molecular mechanisms of potential Cd stress response genes is crucial [8, 9].

Several studies on Cd stress-related genes and mechanisms are available, for instance, the phytochelatin synthase 2 gene (*OsPCS2*) from *Oryza sativa* improved Cd and As stress tolerance by mitigating the accumulation of these heavy metals in rice plants [10]. The F-box protein PP2-B15 and zinc transporter 4 from *Solanum lycopersicum* are indicators of soil Cd contamination as confirmed by transcriptional analyses [7]. Тhe sensitivity of two near-isogenic *Triticum aestivum* lines with differences at the Rht-B1 locus, Rht-B1a (tall wild type, encoding DELLA proteins), and Rht-B1c (dwarf mutant, encoding modified DELLA proteins) to Cd stress was investigated. The results suggest that the Rht-B1c-encoded DELLA proteins enhanced Cd tolerance by participating in the photosynthetic apparatus [11]. Heavy metal ATPase 3 (HMA3), a P_1B2_-ATPase, is a key tonoplast transporter involved in mediating the vacuolar sequestration of Cd to detoxify the intake of this element by plants. The *HMA3* from *Festulolium loliaceum* (*FlHMA3*) plays an important role in Cd^2+^ sequestration in root cell vacuoles, thereby limiting the entry of Cd^2+^ into the cytoplasm and reducing Cd^2+^ toxicity [12]. Vacuolar H^+^-ATPase (V-ATPase) and its subunits play important roles in heavy metal stress response, and the c subunit from *Tamarix hispida* (*ThVHAc1*) confers plants with enhanced CdCl_2_ stress tolerance through WRKY transcription factor and ROS scavenging [5].

V-ATPase is a multi-subunit complex comprising domains V_1_ (600–650 kDa membrane-peripheral domain) and V_0_ (260 kDa membrane-integral domain). The V_1_ domain contains eight different subunits (A–H) and is responsible for ATP hydrolysis, while the V_0_ domain includes six different subunits (a, d, c, c’, c”, and e) and is responsible for proton translocation [13]. Some subunits of the V-ATPase were previously characterized, such as *Malus domestica* A subunit (*MdVHA-A*) [14], *T. aestivum* (RH8706–49) B subunit (*TaVB*) [15], *Pennisetum glaucum* c subunit (*PgVHA-c1*) [16], and *Arabidop*sis thaliana VHA-B, −E, −G, and -a subunits [17]. Therefore, understanding the abiotic stress response function and the mechanism of V-ATPase as well as its subunits sounds important.

*Juglans regia* is a nut tree cultivated worldwide for its nutritious fruits [18], whose production is limited by various environmental stimuli. Studies on the stress response mechanism of walnut trees are currently lacking; therefore, achieving a better understanding of the mechanisms involved in abiotic stress response of *J. regia* is timely and essential [19]. In previous studies, we identified a few candidate genes in walnut involved in stress response, including the G subunit (*JrVHAG1*), which is a known drought-inducible osmotic stress gene and correlated with the ABA (abscisic acid)-signal pathway [19]. In this study, we found that the expression of *JrVHAG1* was induced by CdCl_2_ stress in *J. regia* leaves and roots, and the function of *JrVHAG1* exposed to CdCl_2_ treatment was further verified. Meanwhile, the expression activities of *JrVHAG1* promoter segment and upstream regulatory genes were analyzed, which suggested a potential CdCl_2_ response mechanism of *JrVHAG1* involving in ABA-signal pathway and MYB transcription regulation.

## Results

### Expression of *J. regia* V-ATPase subunits and response to CdCl_2_ and ABA treatments

**Fig. 1:**
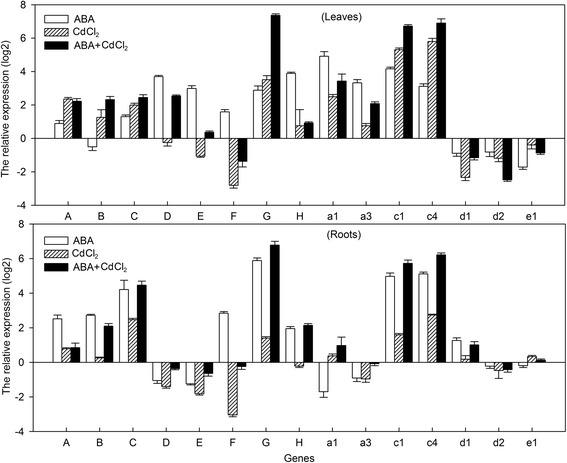
The expression of *JrVHAG1* in *J. regia* under ABA and CdCl_2_ stress. The relative expression level = transcription level under stress treatment/transcription level under control condition (watered well by fresh water at the same time point as stress treatment). Error bars were obtained from three replicates of qRT-PCR

To better understand the role of V-ATPase subunits in abiotic stress response in walnut tree, 15 V-ATPase subunits were identified from the *J. regia* transcriptome in tissues using functional annotation of non-redundant unigenes (NRUs), which were analyzed by blast and denoted as VHA-A, B, C, D, E, F, G (*JrVHAG1*), H, a1, a3, c1, c4, d1, d2, and e1, respectively. Transcription levels of these subunits under CdCl_2_, ABA, and ABA+CdCl_2_ (CdCl_2_ plus ABA) were analyzed by quantitative real time PCR (qRT-PCR) in the roots and leaves of *J. regia*. The results showed that most of these subunits were induced by CdCl_2_ and ABA treatments (Fig. 1). In leaves, the expression patterns upon exposure to ABA, CdCl_2_, ABA+CdCl_2_ could be classified into four groups. VHA-A and e1 had the maximum transcription under CdCl_2_, but had the minimum under ABA stress; While VHA-D, E, F, H, a1, a3, d1 subunits were grouped together based on their expression patterns that were contrary to VHA-A and e1; they hit a peak upon exposure to ABA, but declined to bottom upon exposure to CdCl_2_ stress. The expression levels of VHA-B, C, *JrVHAG1*, c1 and c4 subunits were increased for ABA and ABA+CdCl_2_ stress, whose transcription was up-regulated upon addition of ABA to CdCl_2_ treatments, while the VHA-d2 subunit gene was down-regulated by all the stresses, and the tendency was reverse to the levels of VHA-B, C and G (Fig. 1). Under ABA treatment, the VHA-D, E, G, H, a1, a3, c1, c4 were induced to 7.38-~ 30.20-fold of the control; VHA-B, d1, d2, e1 were suppressed. Under CdCl_2_ stress, VHA-D, E, F, d1, d2 and e1 subunits were down-regulated, among which the F subunit was the least, whereas VHA-G, c1 and c4 were the top induced three genes. When treated with ABA+CdCl_2_, VHA-G, c1 and c4 were induced to 165.42-, 105.42-, 119.43-folds of the control, respectively. Moreover, the transcription of *JrVHAG1* was increased much more obviously by ABA+CdCl_2_ than all other subunits (Fig. 1).

In the roots, the expression profiles of most subunits exposed to ABA, CdCl_2_, ABA+CdCl_2_ were different from those seen in the leaves, excluding VHA-F, d1 and e1 (Fig. 1). According to their transcription patterns, these subunits could also be divided into four groups. Group 1 contained VHA-A, B, F, d1, and d2, whose expression reached a peak when subjected to ABA treatment, while reached the least when subjected to CdCl_2_ stress. The VHA-e1 subunit formed a separate group for its specific expression pattern. VHA-a1 was also by itself in a class as the expression was highest when exposed to ABA+CdCl_2_, while it became the lowest when subjected to ABA. The other subunits were grouped as one family, whose maximum transcription was noticed when treated with ABA+CdCl_2_, while the minimum occurred when subjected to CdCl_2_ stress (Fig. 1). Under ABA treatment, the expression of VHA-C, G, c1, c4 were induced much more obviously than others, and the expression values were 18.55-~ 58.89-fold of the control. The VHA-D, E, a1, a3 were all down-regulated to lower levels compared to the other subunits. Under CdCl_2_ stress, the VHA-F subunit was strongly suppressed. The VHA-C, G, c1, c4 were up-regulated to 2.65-~ 6.65-fold of the control. When treated with ABA+CdCl_2_, VHA-C, G, c1, c4 were strongly induced to a much higher level than other subunits, and the highest transcription level was *JrVHAG1* (110.13-fold of those of the control) (Fig. 1).

### Overexpression of *JrVHAG1* improves plant CdCl_2_ stress tolerance

**Fig. 2:**
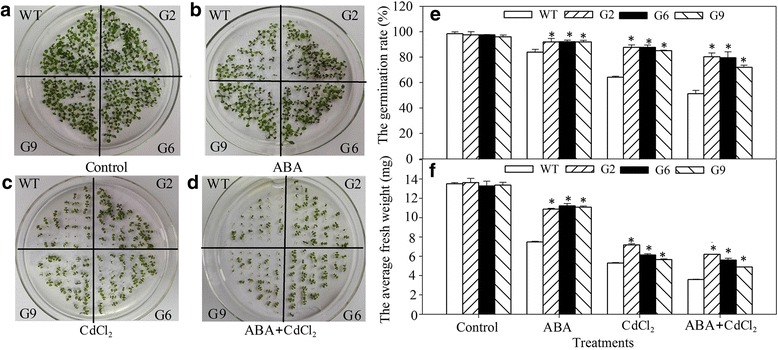
CdCl_2_ stress tolerance analysis of wild type plants and the transgenic Arabidopsis. WT, wild type; G2, G6, G9 are three *JrVHAG1* transgenic lines. **a-d**, germination under normal condition, ABA, CdCl_2_ and ABA+CdCl_2_ treatments, accordingly; **e**, germination percentage of WT, G2, G6, and G9 according to **a**-**d**; **f**, average fresh weight of the germinated seedlings from **a**-**d**. All data are displayed as the mean ± S.D. of three independent experiments. * means the differences between WT and transgenic seedlings were significant (*p* < 0.05)

**Fig. 3:**
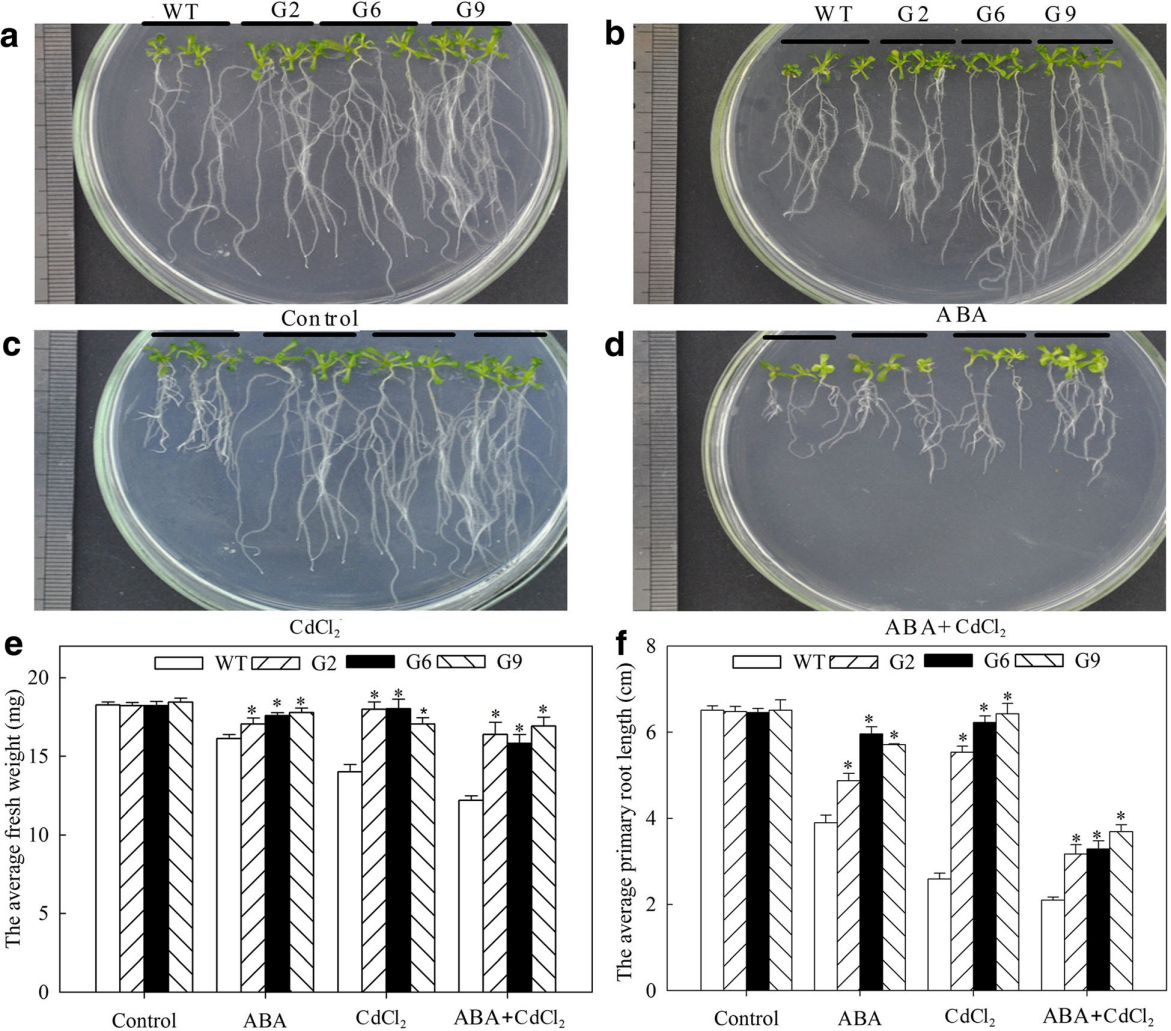
Biomass comparison of WT and transgenic plants under CdCl_2_ and ABA stress. **a**-**d**, the growth of WT, G2, G6, and G9 under normal conditions, ABA, CdCl_2_ and ABA+CdCl_2_ treatments, respectively; **e**, average fresh weight of the seedlings from **a**-**d**; **f**, primary root length according to **a** and **d**. All data are displayed as the mean ± S.D. of three independent experiments, significant differences between transgenic plants and WT were marked as * (*p* < 0.05)

For a complete characterization of the CdCl_2_ stress tolerance of *JrVHAG1* gene, whether it involves the ABA-signal pathway, the germination, biomass, ROS metabolism, and physiological performance of three transgenic lines that overexpression of *JrVHAG1* (G2, G6, and G9) [19] and wild type (WT) Arabidopsis were analyzed when exposed to CdCl_2_, ABA and ABA+CdCl_2_. The results showed that the germination rates of G2, G6, and G9 were significantly higher (*p* < 0.05) than that of WT under the three treatments, which averaged 1.10-, 1.35-, and 1.51-fold increase compared to that of the WT under CdCl_2_, ABA, and ABA+CdCl_2_, respectively (Fig. 2a-e). The average fresh weight of the germinated seedlings of G2, G6, G9 were 1.48-, 1.19-, 1.55-folds higher than those of WT when subjected to the above treatments, accordingly (Fig. 2f). When 8-day-old seedlings of WT, G2, G6, and G9 grown on 1/2 MS were transferred to 1/2 MS with or without ABA, CdCl_2_, ABA+CdCl_2_ and cultured for additional 8 d, the fresh weight and primary root length were similar among the four lines under control conditions. However, the biomass accumulation (fresh weight and primary root length) of the transgenic lines were significantly higher (*p* < 0.05) than that of WT (Fig. 3a-d). The average fresh weight of G2, G6, and G9 lines were 1.26- (CdCl_2_) and 1.34-folds (ABA+CdCl_2_) of that of WT (Fig. 3e). Growth of the primary roots of the WT were severely affected by the three treatments, among which the most serious inhibition was happened on ABA+CdCl_2_ stress (Fig. 3f). These results suggest that overexpression of *JrVHAG1* plays a positive role in the response to CdCl_2_ stress in plants.

**Fig. 4:**
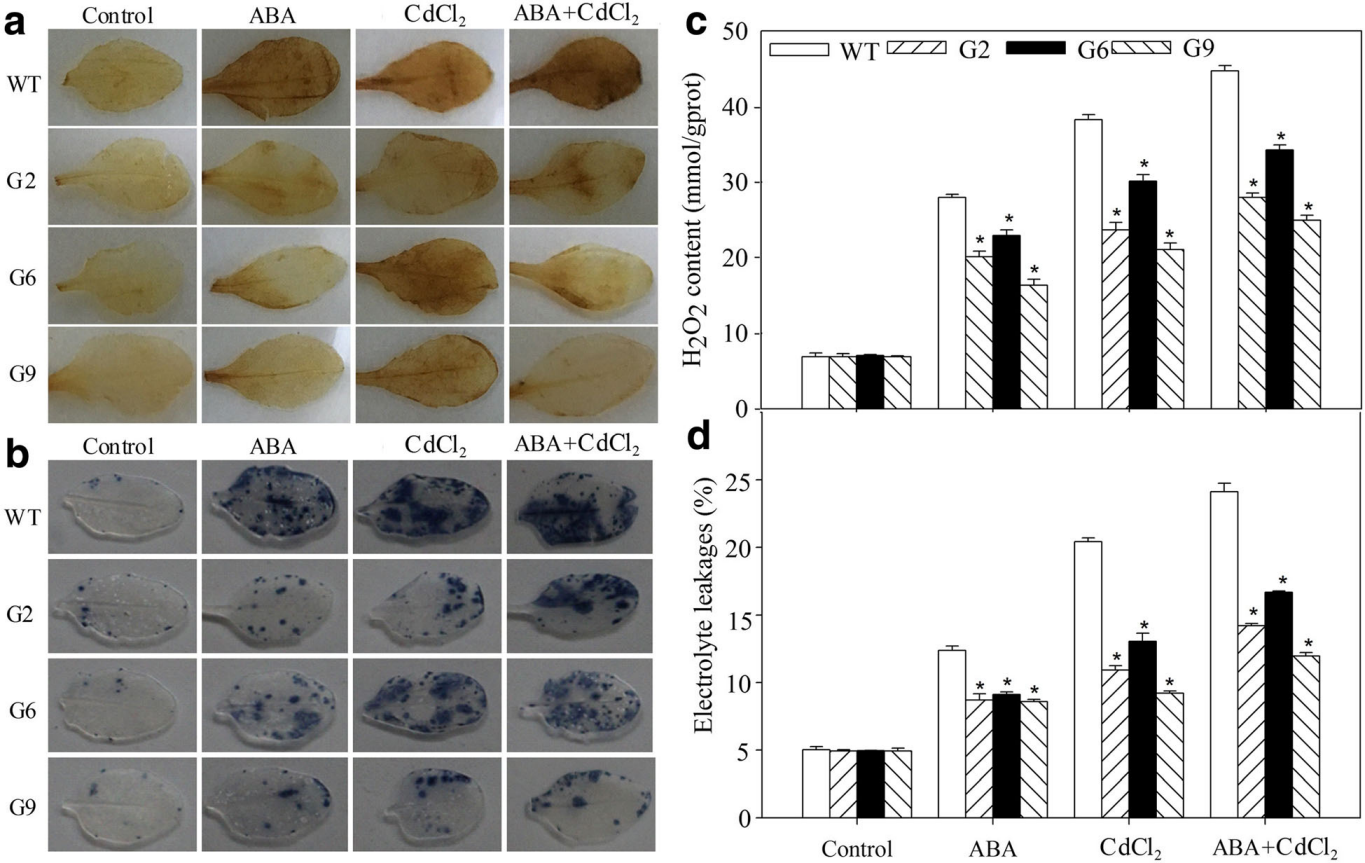
ROS levels in transgenic plants and WT under CdCl_2_ and ABA stress. **a**, DAB staining; **b**, NBT staining; **c**, H_2_O_2_ content; **d**, EL rate. All experiments were repeated at least three times, and approximately 15 samples collected from multiple seedlings in each experiment. All data are displayed as the mean ± S.D. of three independent experiments, significant differences between transgenic lines and WT (*p* < 0.05) were indicated by *

**Fig. 5:**
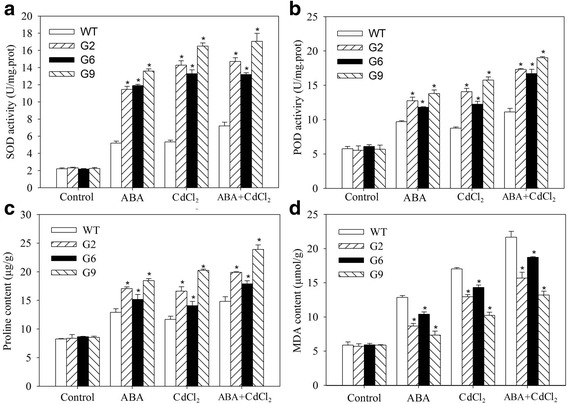
Physiological index analysis of *JrVHAG1* transformed seedlings under CdCl_2_ and ABA stress. **a**, SOD activity; **b**, POD activity; **c**, proline content; **d**, MDA content. Every experiment was repeated three times. All data are displayed as the mean ± S.D. of three independent experiments. * means significant differences (*p* < 0.05) between WT and transgenic seedlings

To further verify the CdCl_2_ tolerance role of *JrVHAG1*, 47-d-old WT and transgenic plants were treated with ABA, or CdCl_2_, or ABA+CdCl_2_. 3, 3′-Diaminobenzidine (DAB) and nitrogen blue tetrazolium (NBT) staining clarified that the WT seedlings were stained deeper than those of G2, G6, and G9 when exposed to all above three treatments (Fig. 4a, b). The hydrogen dioxide (H_2_O_2_) accumulation in the WT plants was also significantly higher than those seen in the transgenic plants (Fig. 4c). Furthermore, the electrolyte leakage (EL) rates of these lines had a trend similar to that as H_2_O_2_. Under ABA treatment, the EL rates of G2, G6, G9 were 69.28%~ 73.57% of that of WT; but when exposed to CdCl_2_, the differences were much more obvious compared to those under ABA stress and they were 45.14%~ 63.98% of that of WT; When treated with ABA+CdCl_2_, the corresponding rates were 49.57%~ 69.13% of that of WT, respectively (Fig. 4d). The superoxide dismutase (SOD) and peroxidase (POD) activities, proline and malondialdehyde (MDA) contents were also supportive of the role of *JrVHAG1* that it could effectively improve plant tolerance to CdCl_2_ (Fig. 5). Upon exposure to ABA, CdCl_2_, ABA+CdCl_2_ stresses, the MDA contents of the tested lines were similar to that of the EL rates and H_2_O_2_ contents, WT had higher values, 1.24-~ 1.75-fold, 1.19-~ 1.67-fold and 1.16-~ 1.64-fold of transgenic seedlings, respectively. However, the SOD, POD and proline levels were contrasting with MDA levels that the SOD, POD and proline levels of three transgenic lines were 2.08-~ 2.75-fold, 1.32-~ 1.60-fold and 1.31-~ 1.45-fold of that of WT, accordingly (Figs. 4, 5).

### Identification and expression activity of *JrVHAG1* promoter

**Fig. 6:**
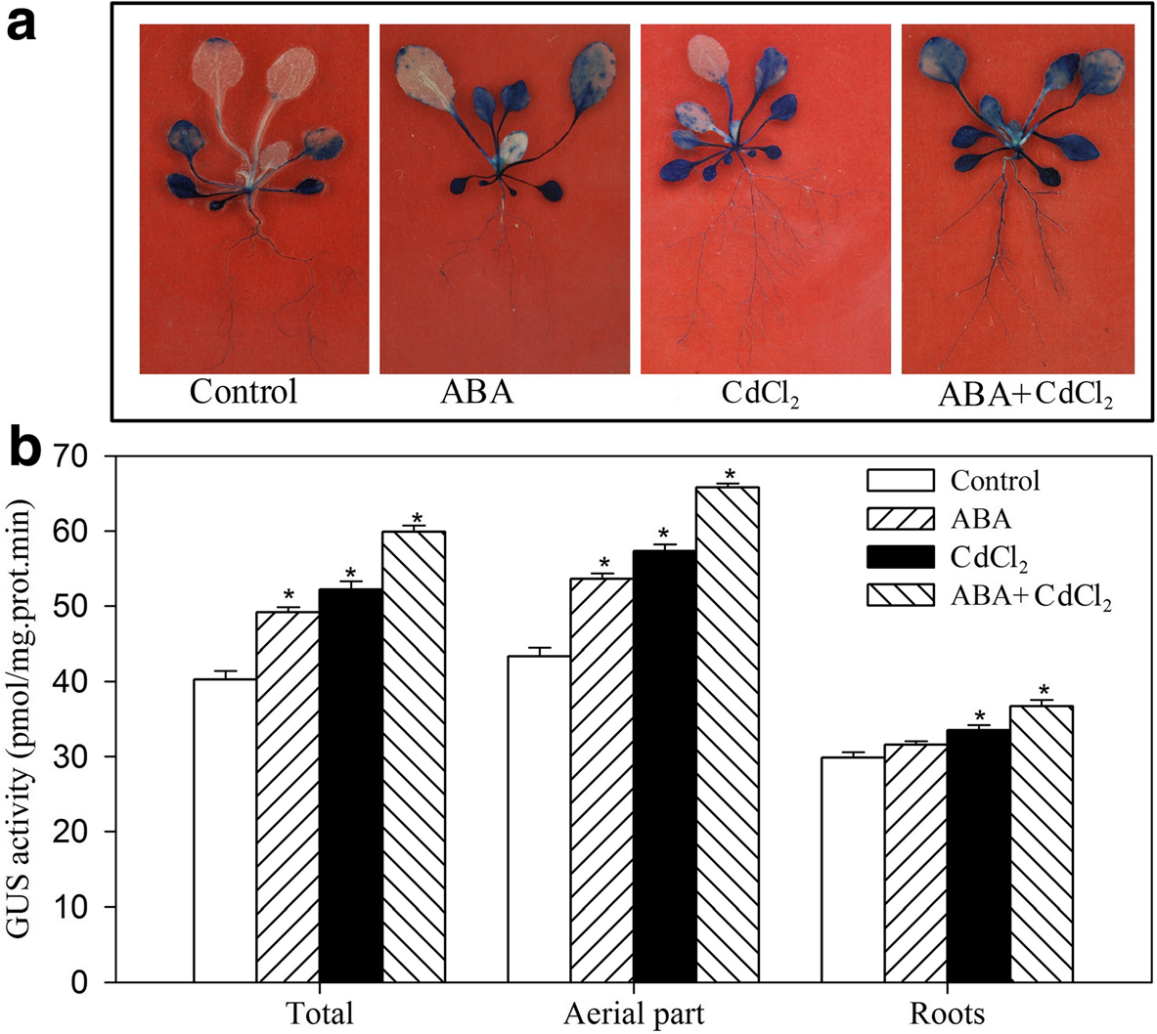
The expression activity of *JrVHAG1* promoter. **a**, GUS staining of the *JrVHAG1* promoter transgenic plants under normal, ABA, CdCl_2_, ABA+CdCl_2_ conditions. **b**, the GUS activities according to **a**. Every experiment was repeated three times. All data are displayed as the mean ± S.D. of three independent experiments. * means significant differences (*p* < 0.05) between the stress conditions and normal condition

A 1200 bp promoter segment of *JrVHAG1* was identified from the *J. regia* genome that was located in the 538,241–534,657 region of the walnut genome (NW017389860.1). The promoter sequence and the relevant elements such as, ARE, LTR, MYBCORE, W-box are shown in Additional file 1: Figure S2. This promoter fragment was inserted into a β-glucuronidase (*GUS*) expression vector pCAMBIA1301, and Arabidopsis plants were transformed using this vector. GUS staining revealed that the promoter caused *GUS* expression in the leaves and roots (Fig. 6). When treated with ABA, CdCl_2_, and ABA+CdCl_2_, the transgenic plants containing this promoter showed increased GUS activity overall in the aerial parts and roots compared to control. The total GUS activities exposed to ABA, CdCl_2_, and ABA+CdCl_2_ stresses were 1.22-, 1.30-, and 1.49-fold higher of that under normal condition, correspondingly, and the differences were significant (Fig. 6).

### Up-stream regulation of *JrVHAG1* involves MYB transcriptional activation

**Fig. 7:**
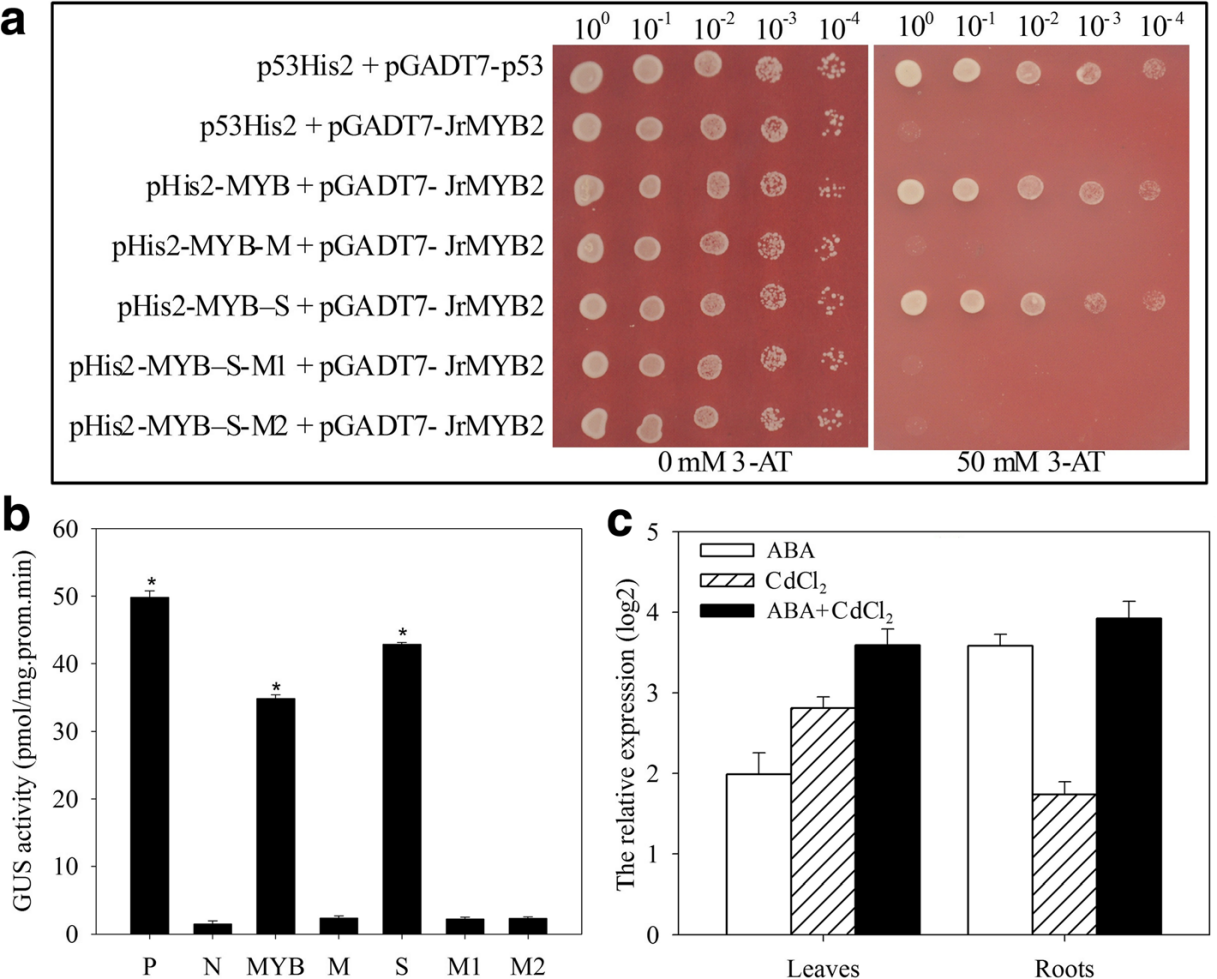
Analysis of the up-steam regulators of *JrVHAG1*. **a**, yeast one-hybrid assay analyses of the upstream regulators of *JrVHAG1*. p53His2 + pGADT7-p53, positive control, pGADT7-Rec2 vector that encodes murine p53 fused with GAL4 AD; p53His2 + pGADT7-JrMYB2, negative control, pHis2 reportor vector that contains the *cis*-acting DNA consensus sequence recognized by p53. The transformants spotted on SD/-His/−Leu/−Trp plates with 0 mM 3-AT were used as positive controls for transformant growth. Positive transformants were further confirmed by spotting serial dilutions (1/1, 1/10, 1/100, 1/1000, 1/10000) onto SD/-His/−Leu/−Trp plates with 50 mM 3-AT. **b**, transient co-transformation of the reportor and effectors into tobacco seedlings, the GUS activities was determined. P, positive control; N, negative control, MYB, M, S, M1, M2 were consistent with a. * means significant differences (*p* < 0.05) between the negative control and others. **c**, the expression of *JrMYB2* under ABA, CdCl_2_, ABA+CdCl_2_ treatments. Every experiment was repeated three times. All data are displayed as the mean ± S.D. of three independent experiments.

To verify the up-stream regulation of *JrVHAG1*, yeast one-hybrid assay was employed to study the interactions between transcription factors (TFs) and MYBCORE in the promoter. It was found that *JrMYB2* binds to the MYBCORE motif, which was confirmed by the interaction between pHis2-MYB-M (mutated MYBCORE), pHis2-MYB-S (the *JrVHAG1* promoter including the MYBCORE motif), pHis2-MYB-M1 (the *JrVHAG1* promoter excluding the MYBCORE motif), or pHis2-MYB-M2 (the *JrVHAG1* promoter including the mutated MYBCORE motif) and *JrMYB2* on the SD (synthetic drop-out medium)/−Trp-Leu-His/50 mM 3-AT (3-amino-1, 2, 4-triazole) solid medium (Fig. 7a). Moreover, the co-transformation of the reportor and effector demonstrated that the GUS activities of the leaves that were transformed by the MYBCORE motif or the promoter fragments containing the MYBCORE motif were similar to that of the positive control, and significantly higher than those of the negative control and mutated reportors. The GUS activities of leaves transformed by effector and the mutated reportor was similar to that of the negative control, further suggesting that *JrMYB2* binds specifically to the MYBCORE motif in the *JrVHAG1* promoter (Fig. 7b).

The results of qRT-PCR analysis of *JrMYB2* clearly showed that *JrMYB2* was induced by ABA, CdCl_2_, and ABA+CdCl_2_ in the leaves and roots (Fig. 7c). In leaves, the transcription of *JrMYB2* was induced by 3.97-, 7.01-, and 12.04-fold of control when subjected to ABA, CdCl_2_, and ABA+CdCl_2_ stresses, and the corresponding gene expression in the roots were 11.96-, 3.34-, and 15.14-fold compared to control, respectively (Fig. 7c). It appears that the expression profiles of *JrMYB2* under these stresses in both tissues are similar to those of *JrVHAG1,* especially when subjected to the same conditions (Fig. 1), indicating that *JrMYB2* may act as an up-stream regulator of *JrVHAG1* to either control *JrVHAG1* or act along with *JrVHAG1* to improve CdCl_2_ stress tolerance of plants.

## Discussion

The V-ATPase regulates and is also self-regulated by various signaling cascades controlling nutrient supply and metabolism [20]. The up-regulation of the V-ATPase subunits is beneficial to plants as part of the heavy metal stress response. For instance, *Cucumis sativus CsVHA-c1*, *CsVHA-c2*, and *CsVHP1;1* are essential elements of the mechanisms involved in the adaptation of cucumber plants to copper toxicity [21]. *T. hispida* c subunit (*ThVHAc1)* was up-regulated by CdCl_2_ stress, and the overexpression could effectively improve the CdCl_2_ tolerance of plants [5]. In a previous study, we demonstrated that the involvement of *JrVHAG1* in drought-inducible osmotic stress correlated with ABA-signal pathway [19], implying the potential response mechanism of *J. regia* V-ATPase and its subunits involve in ABA-signal regulation. To well understand the adaptation mechanism of *J. regia* to heavy metal stress, in this study, we firstly identified 15 V-ATPase subunits and their expression were analyzed by qRT-PCR under CdCl_2_ and ABA treatments. The results showed that the expression of *VHA-B*, *C*, *JrVHAG1*, *c1* and *c4* subunits were similar and induced much more obviously than others exposed to the three treatments, whose transcription was up-regulated more obviously by ABA+CdCl_2_ than single stress of ABA or CdCl_2_ (Fig. 1), suggesting the possibly positive role of *VHA-B*, *C*, *JrVHAG1*, *c1* and *c4* to CdCl_2_ stress and may involve in ABA-signal pathway. To understand rapidly of the prediction, the open reading frames (ORFs) of *VHA-B*, *C*, *JrVHAG1*, *c1* and *c4* were inserted into the pYES2 and transformed into INVSC1, respectively [22]. Then the transgenic yeasts were treated with ABA, CdCl_2_, ABA+CdCl_2_, and compared to the control yeast. The growth abilities of *VHA-B*, *C*, *JrVHAG1*, *c1* and *c4* transformed yeasts were higher than that of the control yeast. The most important is that the *JrVHAG1* transformed yeasts showed the best growth activities under the three treatments, and the differences between *JrVHAG1* transgenic yeast and others were significant under ABA+CdCl_2_ stress (Additional file 1: Figure S1), indicating that the ABA truly promoted the function of *JrVHAG1* in CdCl_2_ tolerance. Considering that the expression of *JrVHAG1* was highly induced by CdCl_2_ stress and was enhanced to a very high level by ABA+CdCl_2_ (Fig. 1), we think *JrVHAG1* was a potential candidate gene for CdCl_2_ stress tolerance and was likely to connect with ABA signal.

To verify the function of *JrVHAG1* in Cd stress, transgenic Arabidopsis lines with high expression of this gene were evaluated. The results showed that the germination rates, biomass accumulations, and protective enzyme activity of the transgenic lines were significantly higher than those of the WT plants; while the ROS generation of G2, G6, and G9 were significantly lower than that of WT (Figs. 2, 3, 4, 5) and perhaps indicating the *JrVHAG1* gene functions in ROS scavenging, suggesting that the expression of *JrVHAG1* could improve plant Cd stress tolerance. These physiological index endowing by *JrVHAG1* under ABA, CdCl_2_ and ABA+CdCl_2_ treatments were similar to other subunits those were verified to be abiotic stress tolerance genes. Such as *M. domestica MdVHA-A* and *Puccinellia tenuiflora PtVHAc*. Overexpression of *MdVHA-A* conferred transgenic tobacco seedlings with enhanced drought tolerance by improving important attributes such as dry weight, fresh weight, MDA, and relative water content [14]. Transgenic *A. thaliana* lines expressing *PtVHAc* exhibited improved tolerance to salt-induced osmotic stress as revealed from the analysis of the fresh weight, root length, silique number, and other parameters [23]. Moreover, ABA is known as a stress hormone that takes part in the integration of signals [24]. Germination and plant growth under heavy metal stress is related to ABA [25, 26]. The current results also revealed that the difference of germination rates and average fresh weight between the transgenic lines and WT under ABA+CdCl_2_ stress was significant than that exposed to CdCl_2_ (Fig. 2), implying that the ABA promotes the function of *JrVHAG1* gene in response to CdCl_2_ stress, which were just similar to other previous reports. For example, after exposure to CdCl_2_ stress, the *S. lycopersicum* Micro-Tom (MT) sitiens ABA-deficient mutant (sit) and its WT counterpart displayed differences in lipid peroxidation, hydrogen peroxide content, and activities of some key antioxidant enzymes such as catalase, glutathione reductase, and ascorbate peroxidase, highlighting the relative importance of ABA in Cd stress response of plants [24]. The basic helix-loop-helix (bHLH) in tartary buckwheat (*Fagopyrum tataricum*) (*FtbHLH3*) was induced by polyethylene glycol 6000 (PEG_6000_) and ABA treatment. Overexpression of *FtbHLH3* in *Arabidopsis* resulted in increased drought/oxidative tolerance and indicates that *FtbHLH3* may function as a positive regulator of drought/oxidative stress tolerance through an ABA-dependent pathway [27]. These results told us that *JrVHAG1* function as a positive CdCl_2_ response gene involved in ABA-signaling pathway.

In plants, transcription factors are important components of stress response pathways involving in many stress-response genes. There are several studies on the function of Cd-responsive genes; however, the upstream transcriptional regulatory pathways that modulate their responses to Cd are less clear [28]. To better understand the Cd stress response mechanism of *JrVHAG1*, the promoter of *JrVHAG1* was identified and the expression activity was tested. The promoter displayed different expression activities in the roots and leaves under normal and CdCl_2_ treated conditions (Fig. 6), which was similar to the expression of *JrVHAG1* upon exposure to CdCl_2_ stress (Fig. 1), indicating the effectiveness of the selected promoter fragment. Further, the up-stream regulator of *JrVHAG1* was identified through yeast one-hybrid assay and co-transient expression experiments. *JrMYB2* was found to specially bind to the MYBCORE motif present in the *JrVHAG1* promoter, and the expression of *JrMYB2* was similar to that of *JrVHAG1* under ABA, CdCl_2_, and ABA+CdCl_2_ treatments (Fig. 7), indicating that *JrMYB2* acts as an up-stream regulator of *JrVHAG1* and could regulate or combine functionality with *JrVHAG1* in CdCl_2_ stress response. This prediction is similar to that in other studies. For instance, *T. hispida ThWRKY7*, the up-stream regulator of *ThVHAc1*, regulates plant CdCl_2_ tolerance and controls the involvement of *ThVHAc1* in plant CdCl_2_ stress response by binding to the WRKY motif present in the *ThVHAc1* promoter [5]. Lin et al. demonstrated that the *Phaseolus vulgaris* ethylene response factor 15 (*PvERF15*), an upstream transcriptional regulator of the Cd metal response element-binding transcription factor 1 (*PvMTF-1*) was identified using a yeast one-hybrid system. It was strongly induced by CdCl_2_ stress and activated the PvERF15/PvMTF-1 transcriptional interaction [28]. These results suggested that the Cd stress response was controlled by up-stream regulators and yeast one-hybrid assay is an effective way to screen the potential regulatory factors. Meanwhile, MYB TFs are members of a large family that have multiple functions in plant growth and development, flavonoid metabolism, and biotic/abiotic stress responses [29]. In *A. thaliana*, the root-specific transcription factor *MYB72* is required for the onset of induced systemic resistance (ISR) and is also associated with plant survival under conditions of iron deficiency [30]. Ectopic expression of orchid (*Dendrobium* sp. XMW-2002-2) R2R3-MYB gene *DwMYB2* in *Arabidopsis* confers hypersensitivity to iron deficiency in the transgenic plants. The ferric-chelate reductase gene, *AtFRO2*, and the iron transporter genes, *AtIRT1* and *AtIRT2*, are consistently up-regulated by the expression of *DwMYB2*, while other potential iron transporters such as *AtIREG1*, *AtFRD3*, and *NRAMP1* are down-regulated, indicating the role of *DwMYB2* in iron transportation impairment [31]. *SbMYB44*, a R2R3-MYB cloned from *Salicornia brachiata* Roxb., was up-regulated in response to salinity, desiccation, high temperature, and treatments with ABA and salicylic acid (SA). Overexpression of *SbMYB44* enhanced the growth of yeast cells under both ionic and osmotic stresses [32]. Considering all these studies and our results, we consider that the *JrVHAG1* gene is an important CdCl_2_ response gene and is involved in MYB transcription pathway.

## Conclusion

The transcription of *JrVHAG1* was highly up-regulated by CdCl_2_ and ABA stress in *J. regia* roots and leaf tissues. Heterologous overexpression of *JrVHAG1* in *A. thaliana* conferred the transgenic seedlings with increased fresh weight and primary root length, higher SOD and POD activities, as well as lower levels of ROS, H_2_O_2_, MDA, and EL rates than those of WT plants exposed to ABA, CdCl_2_, ABA+CdCl_2_ treatments. A 1200 bp promoter fragment containing many *cis*-elements of *JrVHAG1* was isolated and demonstrated to show high expression activity, which was induced by CdCl_2_ and ABA treatments. Yeast one-hybrid and co-transient transformation assays in tobacco showed that *JrMYB2* could specifically bind to the MYBCORE motif in the *JrVHAG1* promoter. Similar to *JrVHAG1*, *JrMYB2* could also be induced by CdCl_2_ and ABA. Considering that the clear responses of *JrVHAG1* and *JrMYB2* to CdCl_2_ and ABA treatments in walnut root and leaf tissues and the CdCl_2_ tolerance conferred by overexpression of *JrVHAG1* in transgenic Arabidopsis, it can be concluded that *JrVHAG1* plays a positive role in Cd tolerance through ABA-signal pathway and involves in MYB transcription regulation.

## Methods

### Plant materials and growth conditions

New branches from 6-year-old grafted ‘Xiangling’ seedlings (a genotype of *J. regia* widely planted in China) were obtained and grafted to 2-year-old stocks of the same ‘Xiangling’. The grafted seedlings were cultivated for 2-years in a greenhouse (22 ± 2 °C, relative humidity 70 ± 5%, illumination cycle 14/10 h) [33], and treated with 50 μM ABA, or 150 μM CdCl_2_, or 150 μM CdCl_2_ plus 50 μM ABA (ABA+CdCl_2_) by watering the roots for 60 h. The roots and leaves were harvested independently and stored at − 80 °C for qRT-PCR analysis. The seedlings watered only with fresh water for 60 h served as control. Every treatment was applied three times and each treatment contained at least 9 seedlings.

### RNA isolation and expression analysis of V-ATPase subunits

V-ATPase subunits were identified from the *J. regia* transcriptome in tissues using functional NRUs, which were analyzed using BLAST tools. The ORFs were screened using the ORF finder tool (https://www.ncbi.nlm.nih.gov/orffinder/), and the primers used for qRT-PCR analysis were designed from the 5′-end of the ORF (Additional file 1: Table S1). Total RNA from each sample was isolated using the cetyltrimethylammonium ammonium bromide (CTAB) method and reverse-transcribed into cDNA [34], which was diluted to 1/10 of the original concentration with sterile water for use as template of qRT-PCR. The 18S rRNA was used as an internal control gene [35]. The 20 μL reaction mixture contained 2 μL cDNA template (equivalent to 100 ng of total RNA), 0.5 μM of each forward and reverse primer (Additional file 1: Table S1), 10 μL of SYBR Green Real-time PCR Master Mix (CWBIO). The qRT-PCR was performed in a CFX96 Touch™ Real-Time PCR Detection System (Bio-Rad Laboratories, Redmond, WA) [33]. The following cycling parameters were applied for amplification: 94 °C for 30 s followed by 44 cycles at 94 °C for 12 s, 60 °C for 30 s, 72 °C for 40 s, and 1 s at 81 °C for plate reading. To ensure the reproducibility of qRT-PCR results, three independent experiments were carried out. The relative expression levels were calculated based on the threshold cycle according to the 2^-ΔΔCT^ method [36].

### Analysis of CdCl_2_ stress tolerance in *JrVHAG1* transgenic *Arabidopsis* plants

The *JrVHAG1* gene was inserted into Arabidopsis plants and three transgenic lines (G2, G6, and G9) with the highest expression level were analyzed [19]. For the seed germination assay, the seeds of WT, G2, G6, and G9 lines were placed on 1/2 Murashige and Skoog (MS) agar medium with 0 (control), or 50 μM CdCl_2_, 3 μM ABA, 50 μM CdCl_2_ plus 3 μM ABA (ABA+CdCl_2_). Germination rate and average fresh weight of the germinated seedlings were recorded after 12 d of sowing under standard growth conditions (24 °C, 70–75% relative humidity, and 14/10 h light-dark photoperiod). For comparing seedling growth, 8-day-old seedlings of WT, G2, G6, and G9 were grown on 1/2 MS agar medium and were transferred to 1/2 MS agar medium with 0 (control), 50 μM CdCl_2_, 3 μM ABA, 50 μM CdCl_2_ plus 3 μM ABA (ABA+CdCl_2_) and grown for another 10 d after which fresh weight and primary root length were recorded. For analysis of ROS accumulation and variations in physiological performance under above-described treatments, 12-day-old seedlings of WT, G2, G6, and G9 were grown on 1/2 MS, transferred to pots containing a mixture of turf peat and sand (2:1 *v*/v) and grown in a greenhouse for additional 5-weeks. The seedlings were then treated with 0 (normal watered, control), or 50 μM CdCl_2_, 3 μM ABA, 50 μM CdCl_2_ plus 3 μM ABA (ABA+CdCl_2_) for 6 d, then the aerial parts were collected to determine ROS generation. Histochemical staining for ROS generation in the four lines was performed using DAB and NBT methods [5, 34, 37]. Determination of physiological indices including H_2_O_2_ content, MDA, and proline levels, activity levels of SOD and POD, as well as EL rate was according to previous studies [19, 38]. Every assay was applied at least three times and each replicate contained at least 30 seedlings.

### Identification and expression analysis of the *JrVHAG1* promoter

The *JrVHAG1* promoter was identified from the walnut genome [39], and amplified by PCR reaction from the *J. regia* DNA. The *cis*-elements in the *JrVHAG1* promoter were analyzed using the PLANTCARE database (http://bioinformatics.psb.ugent.be/webtools/plantcare/html/) [40]. To understand the expression activity of *JrVHAG1* promoter, this promoter was used to replace the *35S* promoter and cloned into a pCAMBIA1301 vector to drive the expression of *GUS* gene (Additional file 1: Figure S2). The recombinant construct was transferred into *Arabidopsis* through *Agrobacterium*-mediated floral dip method [41]. Four-week-old transgenic seedlings were used to study the expression activity and level through GUS activity determination and staining [5, 42] under normal, CdCl_2_ and ABA stress [50 μM CdCl_2_, or 3 μM ABA, or 50 μM CdCl_2_ plus 3 μM ABA (ABA+CdCl_2_)]. Every treatment was replicated three times and each replicate contained at least 30 seedlings.

### Identification of the upstream regulator of *JrVHAG1*

The core sequence of MYBCORE motif is “CNGTTR”, and three MYBCORE elements (“CAGTTG” and “CAGTTA”) were found in the *JrVHAG1* promoter (Additional file 1: Figure S3). Yeast one-hybrid assay was employed to identify the up-stream transcription factors (TFs) capable of recognizing the MYBCORE motif (“CAGTTG” was used in this study). Three tandem copies of “CAGTTG” were cloned into pHis2 vector (pHis2-MYB) (Additional file 1: Figure S4A). MYB TFs were identified from the *J. regia* transcriptome and cloned into pGADT7-Rec2 vector to generate a cDNA library for use in one-hybrid assays [42].

To confirm the interactions between the motif and positive clones, the MYBCORE “CAGTTG” was mutated to “C***CAGG***G” and inserted into pHis2 (pHis2-MYB-M). Fragments of the *JrVHAG1* promoter including the MYBCORE motif (pHis2-MYB-S), excluding the MYBCORE motif (pHis2-MYB-M1), and including the mutated MYBCORE motif (pHis2-MYB-M2) were all cloned into pHis2, respectively (Additional file 1: Figure S4A). The p53His2 construct was used as a control in the yeast one-hybrid assays [5, 42]. All the primers used are listed in Additional file 1: Table S2.

Furthermore, pHis2-MYB/M/S/M1/M2 were independently fused with a *CaMV35S*-46 minimal promoter and cloned into pCAMBIA1301 to drive the *GUS* gene (reportors) expression (Additional file 1: Figure S4C) to confirm the above-described interactions. The ORF of *JrMYB2* (screened TF) was cloned into prokII vector such that it is placed under the control of a 35S promoter (prokII-*JrMYB2*) (Additional file 1: Figure S4B), and can act as an effector. Every reportor was transiently co-transformed with the effector in tobacco leaves using *Agrobacterium*-mediated transformation method, and all co-transformed tobacco leaves were used to measure the GUS activity [42, 43]. Every co-transformation was replicated three times and every replicate contained at least 15 leaves.

### Statistical analysis

All of the data were analyzed using the Statistical Package for Social Science (SPSS) (SPSS, Chicago, Illinois, USA). The differences between the transgenic and WT lines were evaluated using Tukey’s multiple comparison test with the significance level set at *p* < 0.05, and sample variability is reported as standard deviation (S.D.).

## Additional file

Additional file 1: Table S1. The primers used in qRT-PCR analysis. **Table S2.** The primers used in yeast one-hybrid assay and pCAMBIA1301 recombinant vector construction. **Figure S1.** The abiotic stress tolerance analysis of *VHA-B*, *C*, *JrVHAG1*, *c1* and *c4* in yeast expression system compared with empty pYES2 (CK) yeast. The six yeast cultures were independently grown in SC-Ura liquid medium containing 2% (*w/v*) galactose for 20 h at 30 °C to OD_600_ = 0.4, then collecting and adjusting the yeast with Sc-Ura including 2% galactose cultivated to OD_600_ = 1.6 for stress analysis. Yeast cell densities (OD_600_) of *VHA-B*, *C*, *JrVHAG1*, *c1* and *c4* transgenic yeasts and CK were treated with 50 μM ABA, or 150 μM CdCl_2_, or 150 μM CdCl_2_ plus 50 μM ABA (ABA+CdCl_2_) for 0 or 24 h (0 h was set as control) were tested. All data are displayed as the mean ± S.D. of three independent experiments, the significant differences among the six yeasts were compared under the same treatment and indicated by a, b, c (*p* < 0.05), respectively. **Figure S2.** Mapping the reconstruct of *JrVHAG1* promoter that inserted into pCAMBIA1301 vector. **Figure S3.** The promoter and elements of *JrVHAG1* promoter. The MBS means the ‘MYBCORE’ element. **Figure S4.** The binding analysis of *JrMYB2* to the MYBCORE motif. (A) Diagram of the reportor and effector vectors. Three tandem copies of the MYBCORE were inserted into the pHIS2 vector as the reportor construct. (B)The CDS of *JrMYB2* was cloned into pGADT7-Rec2 as the effector construct. The effector and reportor constructs were co-transformed into the yeast strain Y187. (C) Diagram of the reportors and effectors. Triple tandem copies of the W-box were fused with the 35S CaMV-46 minimal promoter and cloned into pCAMBIA1301 for driving the *GUS* gene as the reportor construct. The CDS of *JrMYB2* was cloned into prokII under the control of the 35S promoter as the effector constructs. (DOC 1310 kb)

## Abbreviations

3-AT: 3-amino-1, 2, 4-triazole
ABA: Abscisic acid
bHLH: Basic helix-loop-helix
CTAB: Cetyltrimethylammonium ammonium bromide
DAB: 3,3'′’-Diaminobenzidine
EL: Electrolyte leakage
H_2_O_2_: Hydrogen dioxide
MDA: Malondialdehyde
MS: Murashige and Skoog
NBT: Nitrogen blue tetrazolium
POD: Peroxidase
qRT-PCR: Quantitative Real-Time PCR
ROS: Reactive oxygen species
S.D.: Standard deviation
SD: Synthetic drop-out medium
SOD: Superoxide dismutase
