# Supplementary material for: The walnut JrVHAG1 gene is involved in cadmium stress response through ABA-signal pathway and MYB transcription regulation
Source: BMC Plant Biol. 2018 Jan 22;18:19. doi: 10.1186/s12870-018-1231-7 (PMC5778664; doi:10.1186/s12870-018-1231-7)
Supplement: Additional file 1: Table S1. — The primers used in qRT-PCR analysis. Table S2. The primers used in yeast one-hybrid assay and pCAMBIA1301 recombinant vector construction. Figure S1. The abiotic stress tolerance analysis of VHA-B, C, JrVHAG1, c1 and c4 in yeast expression system compared with empty pYES2 (CK) yeast. The six yeast cultures were independently grown in SC-Ura liquid medium containing 2% (w/v) galactose for 20 h at 30 °C to OD600 = 0.4, then collecting and adjusting the yeast with Sc-Ura including 2% galactose cultivated to OD600 = 1.6 for stress analysis. Yeast cell densities (OD600) of VHA-B, C, JrVHAG1, c1 and c4 transgenic yeasts and CK were treated with 50 μM ABA, or 150 μM CdCl2, or 150 μM CdCl2 plus 50 μM ABA (ABA+CdCl2) for 0 or 24 h (0 h was set as control) were tested. All data are displayed as the mean ± S.D. of three independent experiments, the significant differences among the six yeasts were compared under the same treatment and indicated by a, b, c (p < 0.05), respectively. Figure S2. Mapping the reconstruct of JrVHAG1 promoter that inserted into pCAMBIA1301 vector. Figure S3. The promoter and elements of JrVHAG1 promoter. The MBS means the ‘MYBCORE’ element. Figure S4. The binding analysis of JrMYB2 to the MYBCORE motif. (A) Diagram of the reportor and effector vectors. Three tandem copies of the MYBCORE were inserted into the pHIS2 vector as the reportor construct. (B)The CDS of JrMYB2 was cloned into pGADT7-Rec2 as the effector construct. The effector and reportor constructs were co-transformed into the yeast strain Y187. (C) Diagram of the reportors and effectors. Triple tandem copies of the W-box were fused with the 35S CaMV-46 minimal promoter and cloned into pCAMBIA1301 for driving the GUS gene as the reportor construct. The CDS of JrMYB2 was cloned into prokII under the control of the 35S promoter as the effector constructs. (DOC 1310 kb) [file 12870_2018_1231_MOESM1_ESM.doc]

Table S1 The primers used in qRT-PCR analysis

| Genes | Forward Primer | Reverse Primer |
| --- | --- | --- |
| JrVHA-A | 5′-CTGCAATGTATGAGCTGG-3′ | 5′-AGGCCTCTGAATGCCATC-3′ |
| JrVHA-B | 5′-ACAATCACGACATGGAGG-3′ | 5′-TGTCAATTCCAGACGTTC-3′ |
| JrVHA-C | 5′-ATGGCGACGAGATACTGG-3′ | 5′-CGATCTGACGCCTGATCT-3′ |
| JrVHA-D | 5′-TGCTACTAGAGGTCATGC-3′ | 5′-TCTTGCTTCGATCGAACT-3′ |
| JrVHA-E | 5′-GATCCAGCAGATGGTCAG-3′ | 5′-TACATCATCTTGAGCCTG-3′ |
| JrVHA-F | 5′-AGCTCAGCGCTTATTGCG-3′ | 5′-TAATCATGTTCGCAACAT-3′ |
| JrVHAG1 | 5′-TGCTGACTGCAGAACAAG-3′ | 5′-CCTGTCTCATGCTCAAGC-3′ |
| JrVHA-H | 5′-TCTGGATGATGATGGTCC-3′ | 5′-GCAAGTATCTTACAGCTC-3′ |
| JrVHA-a1 | 5′-GAAGCGATGTGCAGAGAT-3′ | 5′-CTTGAACTCCAAGAGCTC-3′ |
| JrVHA-a3 | 5′-AGCTGGTTCAGCTCATCG-3′ | 5′-GCCTCAAGTTCACCTAGT-3′ |
| JrVHA-c1 | 5′-CGATGAAACGGCACCGTT-3′ | 5′-ACAGGTAATAGGACTTGG-3′ |
| JrVHA-c4 | 5′-TGTATGGGAGCTGCTTAC-3′ | 5′-GAGATGTGCGTAGCCAT-3′ |
| JrVHA-d1 | 5′-TGCTACTAGAGGTCATGC-3′ | 5′-TCTTGCTTCGATCGAACT-3′ |
| JrVHA-d2 | 5′-ACCTTCAACGTTCATGCT-3′ | 5′-CATGTGCTTATAATCCTC-3′ |
| JrVHA-e | 5′-ATGGGATTCTTGGTGACC-3′ | 5′-TCATTCCCCTTCACTCAG-3′ |
| 18S | 5′-GGTCAATCTTCTCGTTCCCTT-3′ | 5′-TCGCATTTCGCTACGTTCTT-3′ |

Table S2 The primers used in yeast one-hybrid assay and pCAMBIA1301 recombinant vector construction

| Genes | Forward Primer | Reverse Primer |
| --- | --- | --- |
| pHIS2 | 5’-GCCTTCGTTTATCTTGCCTGCTC-3’ | 5’-CGATCGGTGCGGGCCTCTTC-3’ |
| pHIS2-MYBCORE | 5’-AATTCCAGTTGCAGTTGCAGTTGGAGCT-3’ | 5’-CCAACTGCAACTGCAACTGG-3’ |
| pHIS2-MYBCORE-M | 5’-AATTCCCAGGGCCAGGGCCAGGGGAGCT-3’ | 5’-CCCCTGGCCCTGGCCCTGGG-3’ |
| pHIS2-MYBCORE-S | 5’-GGAATTCTCAGTTGTTTATCTTCTAA-3’ | 5’-CTCTAGGTTGCATCATTGTCGAGCTCG-3’ |
| pHIS2-MYBCORE-S-M1 | 5’-GGAATTCTTTTATCTTCTAAAATCAAAG-3’ |
| pHIS2-MYBCORE-S-M2 | 5’-GGAATTCTCCAGGGTTTATCTTCTAA-3’ |
| pGAD-JrMYB2 | 5’-AAGCAGTGGTATCAACGCAGAGTGGCCATTATGGCCCATGTATCACGCGAAGAAATT-3’ | 5’-TCTAGAGGCCGAGGCGGCCGACATGTTTTGAACGATGCATGGGAT-3’ |
| pGAD | 5’-CTATTCGATGATGAAGATACCCCACCAAACCC-3’ | 5’-GTGAACTTGCGGGGTTTTTCAGTATCTACG-3’ |
| pCAM-MYBCORE | 5’-AGCTTCAGTTGCAGTTGCAGTTGCCCTTCCTCTATATAAGGAAGTTCATTTCATTTGGAGAGAACACGGG-3’ | 5’-GATCCCCGTGTTCTCTCCAAATGAAATGAACTTCCTTATATAGAGGAAGGGCAACTGCAACTGCAACTGA-3’ |
| pCAM-MYBCORE-M | 5’-AGCTTCCAGGGCCAGGGCCAGGGCCCTTCCTCTATATAAGGAAGTTCATTTCATTTGGAGAGAACACGGG-3’ | 5’-GATCCCCGTGTTCTCTCCAAATGAAATGAACTTCCTTATATAGAGGAAGGGCCCTGGCCCTGGCCCTGGA-3’ |
| pCAM-Seg | 5’- AAGCTTTCAGTTGTTTATCTTCTAA-3’ | 5’-GGATCCCCGTGTTCTCTCCAAATGAAATGAACTTCCTTATATAGAGGAAGGGCTCTAGGTTGCATCATTGTC -3’ |
| pCAM-Seg-M1 | 5’-AAGCTTTTTTATCTTCTAAAATCAAAG-3’ |
| pCAM-Seg-M2 | 5’-AAGCTTTCCAGGGTTTATCTTCTAA-3’ |
| pCAMBIA1301 | 5’-TAGAGTCGACCTGCAGGCAT-3’ | 5’-ATCATCATCATAGACACACG-3’ |
| pROKII | 5’-TTTCATTTGGAGAGAACACG-3’ | 5’-TGCCAAATGTTTGAACGATC-3’ |
| pROKII-JrMYB2 | 5’-ATCGTCTAGAATGTATCACGCGAAGAAATT-3’ | 5’-CGATGGTACCCTATTTTGAACGATGCATG-3’ |
| pCAMBIA1301-promoter | 5’-ATGCAAGCTTGTAACAAAAGATTTTTAGAT-3’ | 5’-TAGCGGATCCATTTTCCTGGAAATAACAAC-3’ |

Fig. S1 The abiotic stress tolerance analysis of *VHA-B*, *C*, *JrVHAG1*, *c1* and *c4* in yeast expression system compared with empty pYES2 (CK) yeast. The six yeast cultures were independently grown in SC-Ura liquid medium containing 2% (*w/v*) galactose for 20 h at 30℃ to OD_600_=0.4, then collecting and adjusting the yeast with Sc-Ura including 2% galactose cultivated to OD_600_=1.6 for stress analysis. Yeast cell densities (OD_600_) of *VHA-B*, *C*, *JrVHAG1*, *c1* and *c4* transgenic yeasts and CK were treated with 50 μM ABA, or 150 μM CdCl_2_, or 150 μM CdCl_2_ plus 50 μM ABA (ABA+CdCl_2_) for 0 or 24 h (0 h was set as control) were tested. All data are displayed as the mean ± S.D. of three independent experiments, the significant differences among the six yeasts were compared under the same treatment and indicated by a, b, c (*p*<0.05), respectively.


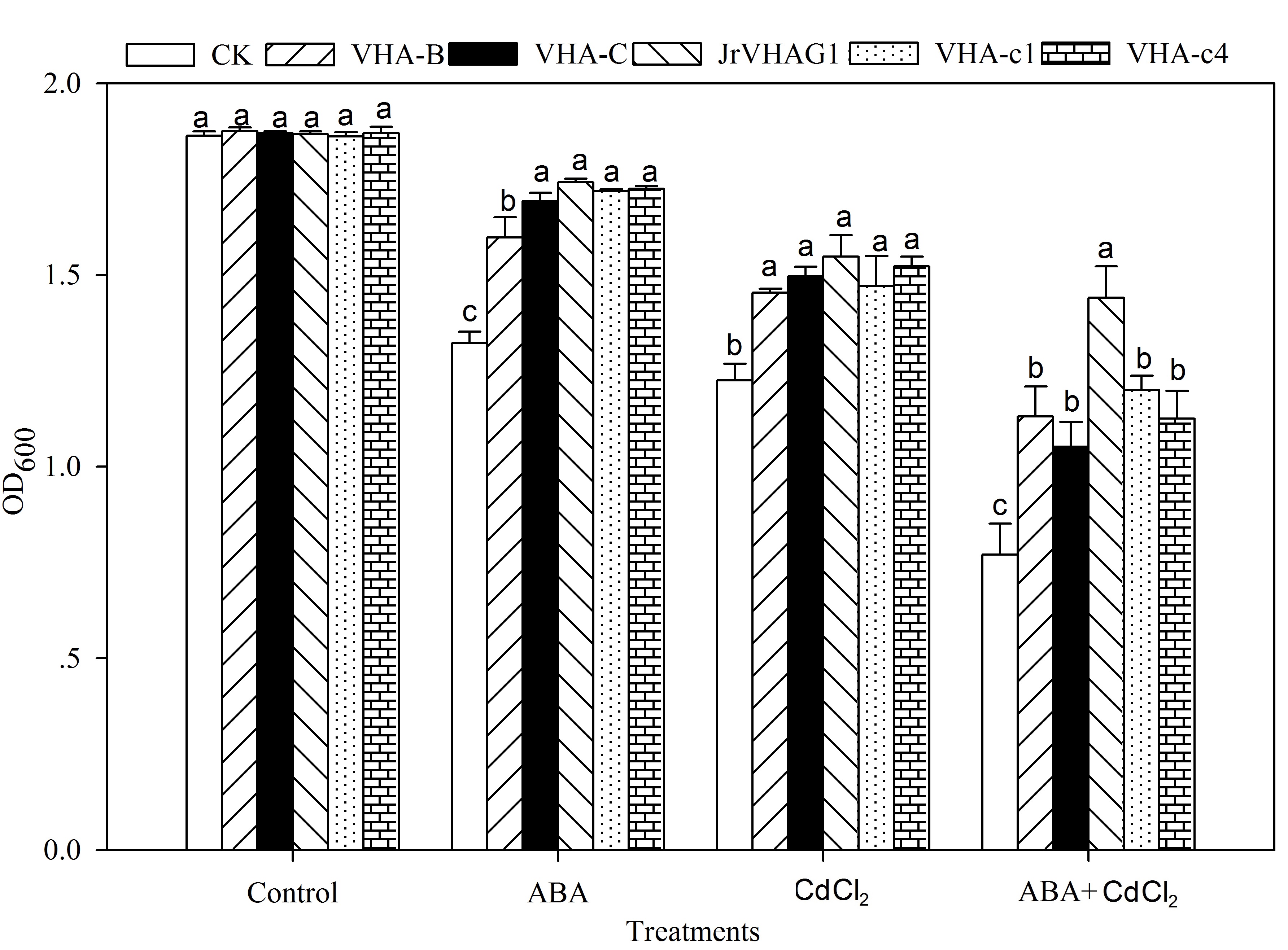


Fig. S2 Mapping the reconstruct of *JrVHAG1* promoter that inserted into pCAMBIA1301 vector


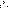

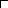

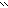

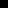

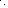


**Hygromycin^r^**

**CaMV 35S**

***JrVHAG1* promoter**

**GUS**

**Nos-T**

**LB**

**RB**

*Xba*Ⅰ

***Nc****o*Ⅰ

Fig. S3 The promoter and elements

of JrVHAG1 promoter. The MBS means


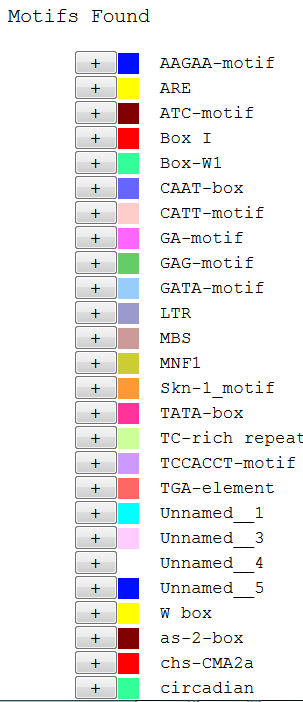

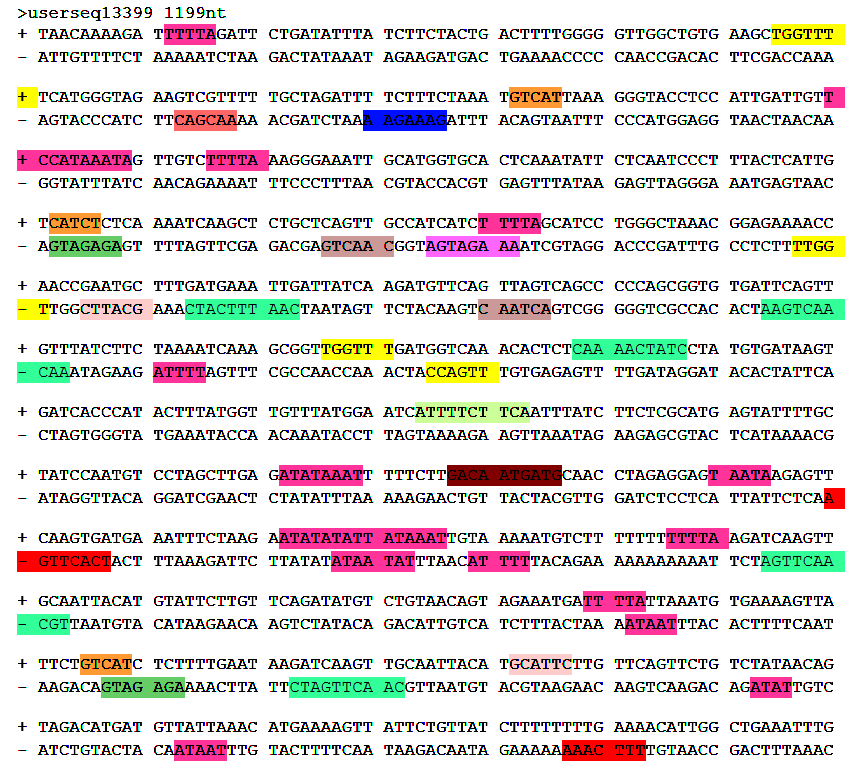

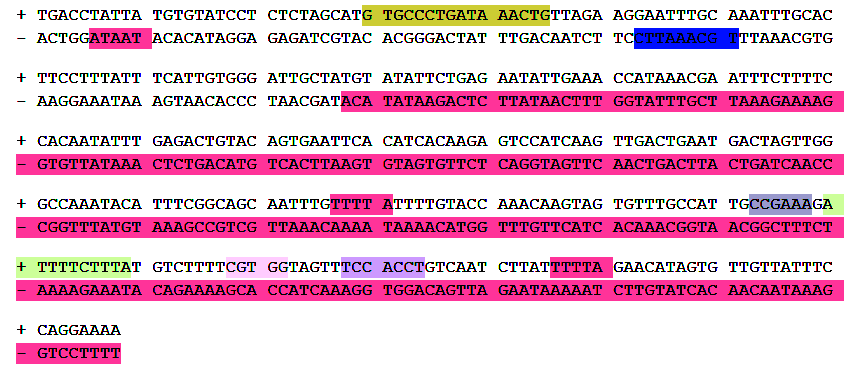


the ‘MYBCORE’ element.

Fig. S4 The binding analysis of *JrMYB2* to the MYBCORE motif. (A) Diagram of the reportor and effector vectors. Three tandem copies of the MYBCORE were inserted into the pHIS2 vector as the reportor construct. (B)The CDS of *JrMYB2* was cloned into pGADT7-Rec2 as the effector construct. The effector and reportor constructs were co-transformed into the yeast strain Y187. (C) Diagram of the reportors and effectors. Triple tandem copies of the W-box were fused with the 35S CaMV-46 minimal promoter and cloned into pCAMBIA1301 for driving the *GUS* gene as the reportor construct. The CDS of *JrMYB2* was cloned into prokII under the control of the 35S promoter as the effector constructs.


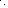

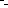

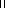


m-Cis×3


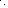

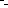

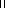


△promoter


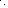

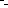

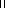


△m-promoter


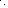

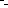

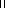


Cis×3

P_mini-HIS3_

*HIS3*

*T_mini-HIS_*_3_

Reportor

GAL4

JrMYB2

Ter


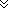

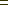

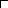

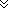


Pro

Effector

A

B


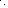

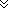

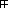


LB

3×element/

promoter flagment

46 bp mini

promoter

GUS

Nos-T

RB

Reportor vector


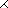

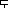


LB

CaMV35S

*JrMYB2*

Nos-T

RB

Effector vector

C
